# The global burden of neonatal hypothermia: systematic review of a major challenge for newborn survival

**DOI:** 10.1186/1741-7015-11-24

**Published:** 2013-01-31

**Authors:** Karsten Lunze, David E Bloom, Dean T Jamison, Davidson H Hamer

**Affiliations:** 1 Boston University School of Medicine, 801 Massachusetts Avenue, Crosstown 2077, Boston, Massachusetts 02118, USA; 2 Department of Global Health and Population, 665 Huntington Avenue, Building I 12th Floor, Boston, Massachusetts 02115, USA; 3 Department of Global Health, University of Washington, 325 9th Avenue, Ste. 359931, Seattle, WA 98104, USA; 4 Department of International Health and Medicine, Boston University Schools of Public Health and Medicine, 801 Massachusetts Avenue, Boston, Massachusetts 02118, USA; 5 Zambia Centre for Applied Health Research and Development, 4649 Beit Road, Lusaka, Zambia

**Keywords:** hypothermia, mortality, neonatal, newborn, prematurity

## Abstract

**Background:**

To provide evidence on the global epidemiological situation of neonatal hypothermia and to provide recommendations for future policy and research directions.

**Methods:**

Using PubMed as our principal electronic reference library, we searched studies for prevalence and risk factor data on neonatal hypothermia in resource-limited environments globally. Studies specifying study location, setting (hospital or community based), sample size, case definition of body temperature for hypothermia, temperature measurement method, and point estimates for hypothermia prevalence were eligible for inclusion.

**Results:**

Hypothermia is common in infants born at hospitals (prevalence range, 32% to 85%) and homes (prevalence range, 11% to 92%), even in tropical environments. The lack of thermal protection is still an underappreciated major challenge for newborn survival in developing countries. Although hypothermia is rarely a direct cause of death, it contributes to a substantial proportion of neonatal mortality globally, mostly as a comorbidity of severe neonatal infections, preterm birth, and asphyxia. Thresholds for the definition of hypothermia vary, and data on its prevalence in neonates is scarce, particularly on a community level in Africa.

**Conclusions:**

A standardized approach to the collection and analysis of hypothermia data in existing newborn programs and studies is needed to inform policy and program planners on optimal thermal protection interventions. Thermoprotective behavior changes such as skin-to-skin care or the use of appropriate devices have not yet been scaled up globally. The introduction of simple hypothermia prevention messages and interventions into evidence-based, cost-effective packages for maternal and newborn care has promising potential to decrease the heavy global burden of newborn deaths attributable to severe infections, prematurity, and asphyxia. Because preventing and treating newborn hypothermia in health institutions and communities is relatively easy, addressing this widespread challenge might play a substantial role in reaching Millennium Development Goal 4, a reduction of child mortality.

## Background

### The global burden of neonatal deaths and its relation to hypothermia

The global under-5 child mortality rate has decreased continuously during the last three decades, from 110 per 1,000 in 1980 to 60 per 1,000 in 2009, and the number of child deaths worldwide each year has decreased from 13.5 million in 1980 to an estimated 7.7 to 8.8 million in 2008 [1-4]. The number of neonatal deaths (newborns dying under the age of 28 days) has also decreased, from 4.6 million deaths in 1990 to approximately 3.1 to 3.6 million in 2009 [3,5]. However, neonatal mortality has declined at a lower rate than child mortality, so the proportion of newborn deaths among all child deaths has been increasing.

Neonatal deaths are unequally distributed around the globe. Half of the world's newborns die at home, and more than 99% of all deaths occur in developing countries, where the average neonatal mortality rate is 33 per 1,000, compared with 4 per 1,000 in high-income countries. Since neonatal deaths account for more than 40% of under-5 mortalities [3], reaching Millennium Development Goal (MDG) 4 will require a substantial reduction in newborn mortality. Although addressing neonatal hypothermia might facilitate this goal, it has so far been a neglected challenge. Maintaining a normal body temperature is a critical function for newborn survival. Newborns achieve this through sophisticated mechanisms of body temperature regulation controlled by the hypothalamus and mediated by endocrine pathways through shivering and non-shivering thermogenesis [6]. However, particularly in premature and low birth weight infants, thermoregulatory mechanisms are easily overwhelmed, leading to metabolic deterioration and direct death from hypothermia or indirect mortality from associated mortalities such as severe infections [7].

The attribution of neonatal hypothermia to indirect or direct causes of newborn death is complex and difficult for several reasons. Most neonatal deaths occur in regions without vital registrations or other reliable data sources. In countries where neonatal deaths are estimated to be highest, mortality data are based on national sample death registration at best [3] and mostly rely on verbal autopsy data-based models with less-than-optimal sensitivity and specificity to correctly identify causes of deaths [8]. Only 2.5% of global neonatal deaths data are based on reliable vital registration systems [9], while 97% derive from systematic estimations or household surveys [10].

Moreover, causes of death are difficult to ascertain in newborns because, especially at the beginning of life, infants exhibit few specific symptomatic reactions to illnesses. Clinical manifestations of various diseases overlap considerably in neonates. Early deaths and those in very small babies are often misclassified due to varying definitions of stillbirth and neonatal deaths, or due to other misreporting of data, such as avoiding filling out death certificates [8].

Globally, severe infections account for an estimated 36% of all neonatal deaths, while problems associated with prematurity account for another 29%, and birth asphyxia makes up 23% (with congenital malformations and a variety of other causes responsible for 19% of neonatal deaths) [3]. While all these causes of death are associated with neonatal hypothermia, the direction of causality is unclear [7]. Although usually infections (mostly sepsis and pneumonia) are listed as cause of death rather than hypothermia, it is unclear whether hypothermia is the underlying cause or the consequence of severe infections. Like severe infections, prematurity is associated with mortality from hypothermia. While only half of the babies born are weighed at birth (and even fewer are of known gestational age), it is estimated that annually 18 million, or 14% of all babies, are born with low birth weight (LBW), half of them in South Asia. LBW infants account for 60% to 80% of neonatal deaths. Birth asphyxia is associated with hypothermia, and prevention or treatment of hypothermia is an important therapeutic principle during and after resuscitation in both developing and developed settings [11,12].

Daily mortality rates for the neonatal period are 30-fold higher than later during infancy [10]. Immediately after birth, an infant is at highest risk of dying, with 25% to 45% of neonatal mortality occurring during the first 24 h [10] and 75% of neonatal mortality during the first week of life [13]. Assuming that early deaths are caused mainly by prematurity and asphyxia, interventions addressing hypothermia management and resuscitation might have a substantial impact on neonatal mortality prevention.

This review analyzes the global epidemiological situation of neonatal hypothermia for the purpose of guiding future policy and research efforts.

## Methods

**Figure 1:**
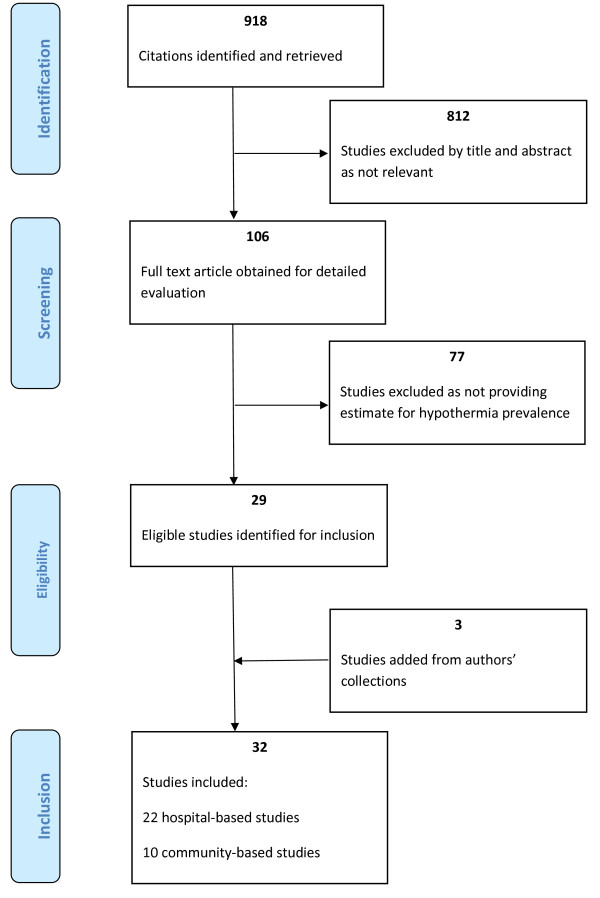
**Study selection for review of global newborn hypothermia**.

We identified studies providing data on the epidemiology of and potential risk factors for neonatal hypothermia, with the search focused on low-income and middle-income countries. This review followed the Preferred Reporting Items for Systematic Reviews and Meta-Analyses (PRISMA) guidelines where applicable [14]. Rather than reviewing only intervention studies on hypothermia and assessing their quality, we reviewed any references with data on the magnitude of hypothermia prevalence as observational outcome or at study baseline. For this analysis, we included individually specified hypothermia threshold definitions for each study in our results summary. We conducted a broad PubMed search of peer-reviewed published papers without date or language restrictions, limited to low-income or middle-income countries. The search (see flowchart in Figure 1) included MeSH terms and keywords, combinations, and snowball searching in references of pertinent papers for related articles. The initial search terms used were (('Hypothermia'[MeSH]) OR 'Body Temperature Regulation'[Majr:NoExp]) AND ('Infant, Newborn'[MeSH] OR 'neonatal'), and included various combinations of these and the terms 'thermoregulation', 'body temperature regulation', and 'heat loss'. The lead author scanned 918 titles and retrieved 360 relevant abstracts. All citations providing newborn hypothermia prevalence estimates or data from which this could be calculated for a low-income or middle-income country (as defined by the World Bank [15]) were deemed eligible for inclusion in this review. Only studies fulfilling criteria determined prior to the literature research were applicable for inclusion into the review: those specifying study location, setting (hospital or community based), sample size, case definition of body temperature for hypothermia, temperature measurement method, and point estimates for hypothermia prevalence. For each identified reference, full-text copies were obtained. Additional references were sought from these articles' reference lists and from additional collection of the authors. We reviewed each pertinent paper in detail and used a systematic coding form to summarize each study's quality measures and further key findings.

## Results

### The prevalence of neonatal hypothermia globally

Newborn hypothermia is ubiquitous, including tropical environments. As summarized in Table 1 by World Health Organization (WHO) region and study setting, we found a total of 31 studies reporting body temperatures in newborns. While 21 studies reporting on hypothermia prevalence were hospital based, we identified 10 community-based studies from Southeast Asia. Three studies from Sarlahi, Nepal, reported hypothermia data on an identical study population [16-18].

**Table 1:** Global epidemiology of newborn hypothermia

Reference	Location	Setting	N	Case definition body temp	Temperature measurements	Hypothermia prevalence	Additional findings
Africa							
Hospital based:							
[28]	Sagamu, Nigeria	University Hospital	150	< 36.5°C	Axillary upon admission	62%	Hypothermia risk highest in the first 24 h of life in preterm babies
[75]	Sagamu, Nigeria	University Hospital	111	< 36.5°C	Axillary upon admission	68%	Hypothermia risk highest in low birth weight babies and those who were not breastfed
[76]	Ibadan, Nigeria	University Hospital	541	< 35.0°C	N/A	45%	Hypothermia more prevalent on admission in infants born outside the hospital than those born at the hospital
[35]	Guinea-Bissau	National Hospital	2,926	< 34.5°C	Axillary within 12 h of birth	8%	Hypothermia of < 34.5°C associated with mortality risk of 4.81 (95% CI 2.90 to 8.00) in first 7 days compared to those without hypothermia
[29]	Kampala, Uganda	Periurban Hospital	300	< 36.5°C	Rectal and tympanic four times within 90 minutes postpartum	79%	Hypothermia incidence increased from 29% at 10 minutes postpartum to 79% at 90 minutes postpartum
[47]	Kampala, Uganda	Periurban Hospital	249	< 36.5°C	Rectal 60 minutes postpartum	46%	Early bathing increased the risk of hypothermia in spite of use of warm water and skin-to-skin care
[25]	Lusaka, Zambia	University Hospital	62	< 36.0°C	Rectal 30 and 120 minutes after delivery	53% and 69%	At discharge after an average of 14 h, hypothermia was still persistent in half of all babies sampled
[26]	Lusaka, Zambia	University Hospital	261	< 36.0°C	Rectal upon admission	44%	Mortality was higher in hypothermic infants than in those who were not hypothermic
[77]	Harare, Zimbabwe	University Hospital	313	< 36.0°C	Axillary	51.4%	
[59]	Harare, Zimbabwe	University Hospital	313	< 36.0°C	Axillary upon admission	85%	
[27]	Ethiopia	Hospital		< 36.0°C	N/A	53%	
Asia							
Hospital based:							
[78]	Iran, different provinces	University Hospitals	1,952	< 36.0°C	Rectal after delivery and four repeats within 6 h of admission to neonatal unit	33.8%	Newborn with low birth weight, prematurity, low Apgar scores, of multiple pregnancies and after cardiopulmonary resuscitation at higher risk for being hypothermic in regression analysis. Hypothermia is associated with an increased risk of neonatal mortality risk (OR = 3.1, 95% CI 1.9 to 5.2) as well as risk of metabolic acidosis, jaundice, respiratory distress, hypoglycemia, and pulmonary hemorrhage.
[33]	Tehran, Iran	University Hospitals	940	< 36.5°C	Rectal upon admission to neonatal unit (mean time after delivery 20 minutes)	53.5%	Hypothermia at birth is associated with an increased neonatal mortality risk (OR = 3.64, 95% CI 1.85 to 7.18), as well as risk for respiratory distress (OR = 2.12, 95% CI 1.53 to 2.93), metabolic acidosis (OR = 2.83, 95% CI 1.74 to 4.59), and jaundice (OR = 2.01, 95% CI 1.45 to 2.79), controlling for weight and gestational age
[32]	Tehran, Iran	University Hospital	900	< 36.5°C	Rectal after delivery and four repeats within 6 h of admission to neonatal unit	53.3%	Low birth weight, low gestational, age environmental temperature, low Apgar score, multiple pregnancy and receiving cardiopulmonary resuscitation increased risk for being hypothermic in regression analysis
[79]	Hangzhou, China	University Hospital	200	< 36.5°C	Axillary after delivery and five times on first, second and third day	52%	Hypothermia risk associated with low birth weight and gestational age
[40]	Kathmandu, Nepal	Maternity Hospital	495	< 36.0°C	Rectal 2 h after delivery	85%	
[80]	Kathmandu, Nepal	Maternity Hospital	82	< 35.0°C	Rectal after delivery	26%	
[60]	Kathmandu, Nepal	Maternity Hospital	100	< 36.0°C	Rectal after delivery	64%	16% of hypothermic infants died within first week of life
[81]	Kathmandu, Nepal	Maternity Hospital	76	< 36.0°C	Axillary after delivery	63%	
[82]	Kathmandu, Nepal	Maternity Hospital	35	< 36.0°C	Continuous axillary and forehead skin probe	72% (Incidence in percent of time being hypothermic from birth to 8 h of life)	
[83]	Mumbai, India	Hospital	206	N/A	N/A	37%	Prevalence 5.9% in infants with kangaroo mother care
Asia							
Community based:							
[19]	Kathmandu, Nepal	Community	12	< 35.0°C	Rectal within 12 h after delivery	91%	
[84]	Kathmandu, Nepal	Community	250	< 36.0°C	Axillary	82%	
[20]	Haryana, India	Community	189	< 35.6°C	Axillary once on first day	11%	Higher prevalence in winter (19%) than in summer (3%); in a secondary analysis applying a case definition of 36.5°C, prevalence was 38%; correlation between room air temperature and body temperature
[21]	Gadchiroli	Community	763	< 35.0°C	Axillary on 8 days during first month	17%	Higher prevalence in winter than in summer
[22]	Uttar Pradesh, India	Community	1,732	< 36.5°C	Axillary 3 to 36 h after birth	43%	Body temperature lower in low ambient temp < 20°C and in newborns with hypothermic mothers
[85]	Dehli, India	Community	32	< 35.0°C	Axillary daily on first 7 days of life	25%	
[39]	Uttar Pradesh, India	Community	148	< 36.5°C	Axillary within 48 h and on days 7, 30 and 60	14%	
[16]	Sarlahi, Nepal	Community	23,240	< 36.5°C	Axillary on 10 days during first month	92.3%	Hypothermia risk highest in the first 72 h of life
[17]	Sarlahi, Nepal	Community	23,240	< 36.5°C	Axillary on 10 days during first month	92.3%	Hypothermia risk highest in preterm babies, females, those breastfed later than 24 h after delivery, and those with hypothermic mothers
[18]	Sarlahi, Nepal	Community	23,240	< 36.5°C	Axillary on 10 days during first month	92.3%	Mortality risk increases by 80% for every 1°C decrease. Mortality risk 6.11 for newborns < 35.0°C compared to those > 36.5°C.
South America							
Hospital based:							
[34]	Recife, Brazil	University Hospital	320	< 36.5°C	Axillary on admission	32%	Hypothermia increased neonatal mortality risk, AOR = 3.49, 95% CI 3.18 to 3.81

These, and an earlier community-based study from Nepal [19], found newborn hypothermia to be almost universal at birth. Overall in community-based studies (all conducted in Nepal or India), hypothermia prevalence ranged from 11% to 92%. Perhaps not surprisingly, lower ambient temperatures [20,21] and cold season [22] were associated with a higher incidence of hypothermia in Indian studies.

However, not only in climatic regions perceived as high risk for hypothermia such as the hills and mountains of Nepal, Northern India, Bangladesh, Pakistan and Bhutan [22] do infants get too cold. Hypothermia is a problem even in tropical countries and warm climates, as was first reported more than fifty years ago [23]. A classic study from Dakar, Senegal found only 5 in 78 babies with a temperature over 36°C [24]. All identified studies from Africa to date are hospital based, report poor newborn practices and high prevalences of newborn hypothermia ranging from 44% to 69% in Zambia [25,26] to 53% in Ethiopia [27], 62% to 68% in Nigeria [28], and 85% in Zimbabwe [29]. Large studies from other countries confirm the global tendency for newborn hypothermia in different climate conditions, from Asia (China) (cited in [30]), [31], the Middle East (Iran) [32,33] and South America (Brazil) [34].

## Discussion

Overall, the prevalence of hypothermia in hospital-based studies ranges from 32 to 85%, with the exception of a low outlier (8% in Guinea Bissau [35]). This wide range might in part be attributable to the varying case definition of hypothermia across studies, ranging from 35.0°C to 36.5°C, and in part to the climatic environment and its seasonal variations discussed below.

We caution that the direct comparability of prevalence data from these hospital-based studies are limited by selection bias, as study populations often represent a high-risk patient cohort and might not be representative of the local population. Data comparability is further limited by the heterogeneity in case definition of hypothermia, which ranges from as low as 35.0°C to the current WHO standard of 36.5°C body temperature, measured with homogeneously temperature measurement methods across studies. Furthermore, prevalence data are confounded by various covariates inconsistently present across studies, such as environmental temperatures and seasonality, the newborns' maturity and age, or maternal factors.

### Risk factors for hypothermia

Various studies identified several risk factors for newborn hypothermia, which we categorize as follows.

#### Environmental

Termed 'contextual' by other authors [36]; initially, an infant's body temperature is associated with maternal temperatures [37]. Several studies have confirmed the intuitive association with environmental temperatures and with the cold seasons. The Gadchiroli trial, with an overall hypothermia prevalence of 17%, showed variations from summer (14.8%) to winter (21.5%) [21,38]. Other studies from Haryana in Northern India recorded an overall hypothermia prevalence of 11%, ranging from 3% in the summer to 19% in winter [20]. In Uttar Pradesh, hypothermia was detected in 14% (n = 148) and was found to strongly correlate with environmental temperature [39]. Another study from the same state found a higher rate of 45%, which likewise was correlated with environmental temperatures and varied considerably over the seasons, ranging from 70% during winter to 20% during summer [22]. Studies from Nepal suggest that the higher prevalence of hypothermia in hospitals during winter months can successfully be addressed through staff training of early drying, wrapping, and breastfeeding [40,41]. The Sarlahi trial found that while even in the hottest season of the year almost one-fifth of infants were hypothermic [16], the risk of moderate-to-severe hypothermia further increased by 41% for each 5°C decrease in ambient temperature [17].

#### Physiological

While newborns of all gestational ages are at risk of losing body heat after birth, premature and small babies are particularly vulnerable due to their physiologic disadvantages. A newborn's thermal regulatory mechanisms are highly sophisticated, but particularly in babies born prematurely easily overwhelmed [7]. Neonatal anatomic characteristics add to the metabolic burden of increased energy requirements: term babies have a 2.7 times greater body surface and preterm babies an up to 4.0 times greater surface per weight than adults.

Several conditions of immature thermal regulation, such as LBW, prematurity, intrauterine growth restriction, and asphyxia (with heat loss due to lack of oxygenation and, where attempted, during reanimation efforts) during birth are significantly associated with an abnormal low body temperature [31,32,40,42,43]. Hypoglycemia is an important contributor to hypothermia [44], and vice versa: it maintains a vicious circle, which leads to feeding weakness, weight loss and finally increased mortality, which first was shown in studies in the 1950s and 1960s [45]. Breastfeeding therefore treats hypothermia not only through bonding with and warming through the mother, but also by replenishing a newborn's glucose levels.

#### Behavioral

Early bathing contributes significantly to heat loss and increases the incidence of hypothermia in cold climates [46] and even in a warm environment [47] and should be postponed until at least after the first 6 h of life, and possibly longer. It is, however, a widespread practice even in high-risk environments [48-51].

Massage and oil applications to clean the child early after birth continue to be a widespread tradition [40]. Evidence for the influence of massage and oil application on hypothermia is contradictory. While suggesting protection from hypothermia [40] and against nosocomial infections in preterm very low birth weight infants [52], it has also been shown in other studies to have detrimental effects on the skin as a protective barrier [53] and to lead to heat loss [54].

#### Socioeconomic factors

An infant's low body temperature is also associated with having a young and inexperienced mother, coming from a family with low socioeconomic status [55], or being born to a mother who already had multiple births [32].

While some of these physiologic risk factors have been documented decades ago, awareness of the risks associated with hypothermia, as indicated in a multinational survey [56] and another one from India [57], indicating that healthcare professionals have limited knowledge of the diagnosis and management of newborn hypothermia. Facilities in resource-limited environments rarely have sufficient capacity to address thermal protection. In a recent study in Zambia, we found that health centers are not well prepared to provide thermal protection, with only very few equipped with heat control for the delivery room (7%) or a neonatal warmer (9%) [58].

### Associations of hypothermia with newborn morbidity and mortality

Several studies investigated the association between neonatal hypothermia and associated mortality risks. In our review, case fatality rates (CFR) for newborn hypothermia globally range from 8.5% to 52% [21,26,34,59,60]. A study from India that included only hypothermic babies specifically investigated morbidities and mortalities and found CFRs that ranged from 39.3% for mild hypothermia to 80% for severe hypothermia. This study demonstrated a dramatic effect of comorbidities and confirmed that hypothermia has a much worse outcome when associated with other newborn problems. Fatality rates increased to 71.4% with hypoglycemia, 83.3% with hypoxia, and 90.9% with shock [61].

However, the above-cited studies do not sufficiently control for potential confounders of the effect of hypothermia on mortality and thus provide implausibly high CFRs in the context of high hypothermia prevalence particularly in community settings. Furthermore, these studies reflect the higher risk of hospital populations selected for these studies, and their CFRs are therefore not applicable to community settings. Yet, hypothermia has been shown to be associated with mortality in a community setting. A community-based study conducted in Sarlahi, Nepal found that mortality increased by approximately 80% for every degree Celsius decrease in first observed axillary temperature and that relative risk of death ranged from 2 to 30 times within the current WHO classification for moderate hypothermia, increasing with greater severity of hypothermia [18].

The evidence on the effect of thermal protection on morbidity and mortality is currently limited. Skin-to-skin care has been shown to substantially reduce neonatal mortality among preterm infants born in facilities [62], but its effectiveness for infants born at term and in communities is less clear. In Zambia, we recently showed that training traditional birth attendants in newborn care emphasizing simple thermal protection (wiping the newborn dry and wrapping the dried infant in a separate piece of cloth), along with resuscitation and early treatment of possible sepsis where indicated, reduced mortality rates at day 28 after birth by 45% [63].

Whether wrapping the newborn in plastic (polyethylene or vinyl) bags is sufficient for thermal protection in low birth weight and premature infants remains controversial. While one recent study failed to show an effect of wrapping on hypothermia rates [64], others found that this approach effectively raised body temperature [65,66] and did so more quickly than radiant heaters [67,68], however did not reduce mortality [69].

### Rethinking and redefining neonatal hypothermia

Our and other reviews [36,70] suggest that the burden of hypothermia is still highly prevalent including in tropical countries, practices contributing to heat loss in the newborn are still deeply rooted in many cultures and are difficult to change, technologies adapted to resource-poor environments are still at a developmental stage, simple thermoprotective interventions and behaviors are insufficiently practiced, providers and caretakers lack an understanding of the problem, and adverse health outcomes continue to take their toll in morbidity and mortality in newborns. There is thus a critical need for researchers and policy makers to take on the challenge of newborn hypothermia and address it within a larger framework of maternal and child health programs.

Qualitative studies from Africa and South Asia suggest that delivery and newborn care practices contributing to heat loss are still common globally. Various cultural and sometimes economic barriers often interfere with implementing simple steps to prevent hypothermia. Heating the birth place is costly for families in resource-poor countries [51], and drying and wrapping the baby is often not a priority when the mother needs attention after delivery [50]. In Ghana, for example, the practice of bathing newborns immediately after delivery is sometimes rooted in concerns about 'ritual pollution' [71] or the belief of helping the baby sleep and feel clean, and reducing body odor in later life; attitudes that informants felt would be difficult to change [72] and which need to be taken into account when programming for behavior change.

### Major gaps in our understanding of neonatal hypothermia

Since in most parts of the world temperature is not measured and recorded in most newborns immediately after birth, the epidemiological picture of hypothermia and its clinical consequences is yet incomplete. Hypothermia is believed to be a common problem not only in developing countries, but also in formerly socialist countries [56]. There are few studies from current lower-middle-income countries.

To the best of our knowledge, no population risk attributable to hypothermia has been published yet. There is no consistent definition of normal newborn body temperature, and consequently data that would allow for pooled risk estimates for newborn hypothermia are still incomplete. Temperatures have been shown to vary widely in healthy newborns [73], and standard medical textbooks disagree on the lower normal limit, ranging from 35.5 to 36.5°C, as well as the normal upper level, citing values from 37.0 to 37.9°C [74].

Standard randomized controlled trials to define temperature thresholds associated with adverse health effects (morbidity as well as mortality) and to quantify the contribution of hypothermia to neonatal mortality as specific cause of death have not been conducted. In fact, those studies might be methodologically impossible to undertake, or at least ethically problematic, because the detection of hypothermia prompts therapeutic intervention and thus artificially reduces the associated mortality risk.

Given these limitations, standard measurements of body temperature could be included in newborn studies to complement available epidemiologic data. Newborn trials conducted for other reasons that include data on newborn body temperature could facilitate further investigation of the association of newborn hypothermia with morbidities and disease-specific mortalities. Axillary measurements with standard digital thermometers are inexpensive and can easily be incorporated into most newborn care clinical guidelines and study protocols.

Currently, the best available data come from trials with hypothermia-unrelated interventions providing temperature data, such as the Sarlahi trial in Nepal [18]. Further studies on the mortality and morbidity risks posed by newborn hypothermia are warranted, particularly in sub-Saharan Africa, to refine the current WHO classification scheme for hypothermia that, as has been suggested, might have to revised into narrower categories to more appropriately reflect the overall mortality-hypothermia risk relationship [18,70].

Further research is needed to understand the magnitude and perception of the problem as well as the feasibility and effectiveness of thermoprotective interventions for newborn morbidity and mortality. Methodologically sound hospital-based and community-based studies are required to understand the problem in sub-Saharan Africa specifically. These studies will ideally include potential confounders and mediating factors that have largely not been adequately addressed so far, such as maternal temperature, environmental conditions, and sociocultural contexts and their association with newborn hypothermia.

Various international neonatal advocacy alliances have included thermoprotection strategies in their newborn health programming, including the Healthy Newborn Partnership (Save the Children), Partnership for Safe Motherhood and Newborn Health (WHO), and the Child Survival Partnership (UNICEF). Nevertheless, hypothermia remains a major challenge for newborn survival. Globally, progress has been particularly slow in improving survival in infants less than 7 days old [10]. This might be attributable to the delivery gap in developing countries for interventions addressing early causes of death such as preterm birth and asphyxia, both of which have worse outcomes in the presence of hypothermia.

More than a quarter of a century ago, a study from Senegal reported: 'Deaths [from hypothermia] seem easy to avoid. Purchasing blankets, putting the newborn babies with their mother and not in cots, prohibiting unnecessary washing of the babies, and supplying maternity hospitals with solar water heaters are all easy steps which could greatly reduce this problem. These improvements are gradually being introduced in the maternity hospital we surveyed though it is proving very difficult to persuade elderly auxiliary midwives not to remove the vernix caseosa by thorough washing, this being part of a strong tradition. Health workers in dispensaries should also be aware of the problem of neonatal hypothermia. We frequently see at our clinic mothers with young infants whose growth seems unsatisfactory. They usually complain about not having enough milk. If the infant is lightly clothed and his rectal temperature is well below normal, he may be expending too much energy on thermogenesis. Advising such mothers to use warmer clothing for their babies is usually enough to make them put on weight. Artificial feeding would be wholly inappropriate in such conditions and could even prove fatal' [24]. As our review suggests, most of these statements and easy remedies still hold true today in most of the world.

## Conclusions

Although hypothermia is a direct cause of death only in a small proportion of newborn mortality, it is closely associated with mortality from common causes of newborn deaths such as severe infections, prematurity, and asphyxia. Neonatal deaths mostly occur at home in low-income countries with weak health systems, where the perception of hypothermia as a risk to the newborn is currently insufficient even among health professionals.

Improvement of practices and services, even (and particularly) where health systems are weak, must focus on the poorest, highest-mortality countries, and on the time of greatest risk: birth and the first days of life. MDG 4 can only be met with a significant decrease in neonatal mortality. We propose that understanding, managing, and significantly mitigating the global burden of hypothermia, a largely understudied risk factor for neonatal survival, might be relatively simple and contribute substantially towards reaching MDG 4.

## Abbreviations

CFR: case fatality rates; LBW: low birth weight; MDG: Millennium Development Goal; WHO: World Health Organization.

## Competing interests

The authors declare that they have no competing interests.

## Authors' contributions

KL and DTJ designed the study. KL, DEB, and DHH conceived the search protocol. All authors contributed to the analysis and interpretation of data. KL drafted the manuscript. All authors revised it critically for important intellectual content, and have given final approval of the version to be published.

## Pre-publication history

The pre-publication history for this paper can be accessed here:

http://www.biomedcentral.com/1741-7015/11/24/prepub
